# Design and Characterization of an Antimicrobial Biocomposite for Wound Dressings

**DOI:** 10.3390/ma17194705

**Published:** 2024-09-25

**Authors:** Leslie Becerril-Serna, Blanca Rosa Aguilar-Uscanga, Mario Flores-Soto, Josué Raymundo Solís-Pacheco, Erick Omar Cisneros-López

**Affiliations:** 1 Centro Universitario de Ciencias Extactas e Ingenierías (CUCEI), Universidad de Guadalajara, Blvd. Gral. Marcelino García Barragán 1421, Guadalajara 44430, Mexico; leslie.becerril0680@alumnos.udg.mx (L.B.-S.); blanca.aguilar@academicos.udg.mx (B.R.A.-U.); 2 Jefatura de Investigación, Universidad del Valle de Atemajac (UNIVA), Av. Tepeyac 4800, Zapopan 45050, Mexico; 3 Centro de Investigación Biomédica de Occidente (CIBO), Instituto Mexicano del Seguro Social (IMSS), Sierra Mojada 800, Guadalajara 44340, Mexico; mariosoto924@yahoo.com.mx

**Keywords:** biocomposite, antimicrobial, physico-mechanical properties, wound dressings

## Abstract

Skin wounds, due to their high vulnerability to infections, represent a significant public health issue. These wounds are not only disabling but also entail costly treatments and slow recovery. Consequently, it is crucial to implement new treatments based on bioactive and natural antimicrobial compounds utilizing fibers, polymers, hydrocolloids, and hydrogels to control potential infections and promote wound healing. This study aimed to develop a biocomposite with antimicrobial activity for the treatment of skin wounds, using sodium alginate, bamboo fiber, and a natural antimicrobial as ingredients. The physico-mechanical properties (Young’s modulus, tensile strength, elongation at break, moisture absorption, and water vapor permeability) and antimicrobial activity against *Escherichia coli*, *Staphylococcus aureus*, and *Staphylococcus hominis* were determined. The results demonstrated that the designed biocomposite possesses adequate physico-mechanical properties, such as flexibility, strength, and water absorption capacity, in addition to exhibiting antibacterial activity, making it suitable to be used as a dressing in wound treatment.

## 1. Introduction

Infections resulting from burns are a significant public health issue, necessitating the development of effective treatments to prevent infections caused by pathogenic microorganisms such as *Staphylococcus aureus*, *Pseudomonas aeruginosa*, *Escherichia coli*, coagulase-negative staphylococci (predominantly *S. epidermidis*), and *Enterococcus* spp. These infections pose a risk to patients with underlying conditions and also delay the healing and re-epithelialization processes [1]. In 2021, the reported global expenditure on materials and dressings for skin wounds, including burns, was approximately USD 20.4 billion. Among these materials, biopolymers of natural origin have demonstrated high efficiency and biocompatibility in treating infections and promoting wound healing, significantly reducing patient recovery time [2].

Currently, most burn and skin wound therapies involve dressings, patches, and gauzes that protect against mechanical injuries but are made from slow-degrading, fossil-based materials. These materials can hinder exudate absorption and disrupt the optimal wound recovery environment. Additionally, those with antimicrobial properties often contain synthetic antibiotics, which may pose health and environmental risks and contribute to resistance development [3,4].

Most of these films are based on biodegradable polymers, defined by the American Society for Testing and Materials (ASTM) and the International Organization for Standardization (ISO) as materials that decompose into CO_2_, methane, water, inorganic components, or biomass due to microbial activity [5]. Advances in biotechnology have enabled the synthesis and production of new plastic materials with the necessary mechanical properties for biomedical use. While the properties of biomaterials vary depending on their chemical composition, source, and synthesis methods, it is essential that their environmental and biodegradable characteristics remain uncompromised. However, these materials must not degrade significantly during their intended use, in this case, the time required for wound healing, and only decompose under specific conditions designed for biodegradation (i.e., composting) [5]. Furthermore, any degradation byproducts should not induce cytotoxicity. In this sense, biomaterials such as polysaccharides have demonstrated an adequate environmental stability for biomedical applications [6].

Among these materials, alginates are particularly notable for their extensive use in wound healing research. Sodium alginate-based biocomposites offer adaptable physical properties to the material, including rigidity, flexibility, and porosity, among others. Due to their chemical composition, these polysaccharides [6] have been utilized to create medical composites, such as an antimicrobial gauze made from sodium alginate and glycerol with a tannic acid coating, showing > 95% reduction in viable colonies of *E. coli* and *S. aureus*. Most antimicrobial agents are immobilized on their surface through entrapment and adsorption techniques, enabling the prolonged release of bioactive compounds [7]. 

A key aspect of effective wound treatment is controlling infections and promoting healing. Common antimicrobial agents including silver, iodine, and chlorhexidine have broad-spectrum inhibition but come with drawbacks like cytotoxicity and high costs [8]. 

In contrast, some biobased materials have demonstrated no cytotoxic effects when interacting with cells, meaning that they do not damage or kill cells, which is critical in biomedical applications. Therefore, antimicrobial agents derived from organic compounds, such as vegetable oils, animal products, and plant extracts like turmeric, honey, and bacteriocins, among others are promising alternatives for wound treatment. Some of these have been used for wound healing since pre-Hispanic times, as their natural components facilitate formulations that promote collagenation, angiogenesis, epithelialization, and bacterial growth control [9]. 

These agents remain a viable alternative to traditional inorganic agents like silver nanoparticles, which at certain concentrations interfere with cell viability and hinder the crucial regenerative mechanisms for wound healing. Their mechanism of action against pathogens commonly includes the destruction of membranes and interactions with the DNA of microbes [6]. Given these characteristics, antimicrobial biocomposites using sodium alginate and glycerol with tannic acid coatings have been developed for medical applications [10,11]. Other materials, such as hydrophilic hydrogel and cathechol-based biopolymers, have demonstrated antimicrobial and anti-biofilm properties [12]. Alginate hydrophilicity and surface porosity allow for the development of films that serve as supports for the controlled administration and release of therapeutic substances and immobilized antimicrobial agents [13]. Additionally, combining alginates with bamboo fibers, known for their mechanical properties, can reduce material costs while utilizing an environmentally friendly, biodegradable, and biocompatible material [14].

When selecting materials for dressings based on biopolymers, biocompatibility must be prioritized, especially for polymers interacting with wounds and influencing cellular functions. This study aimed to design, formulate, develop, and characterize a polymeric biocomposite based on renewable natural materials, with optimal mechanical and antimicrobial properties for potential wound treatment application.

## 2. Materials and Methods

The materials used for the biocomposite were sodium alginate (Sigma Aldrich, St. Louis, MO, USA), bamboo fiber cloth (Biotec Bambus, Zapopan, Mexico) (BFC) as mechanical reinforcement, glycerol (Meyer’s, Chemnitz, Germany), and an antimicrobial extract produced by *Lactiplantibacillus plantarum* LH05. The indicator strains used for the inhibition tests to evaluate antimicrobial activity were *Staphylococcus aureus*, *Staphylococcus hominis*, and *Escherichia coli*. The strains were provided by the Human Milk Research Laboratory (Guadalajara, Mexico) at the University Center for Exact Sciences and Engineering (CUCEI)—University of Guadalajara (UDG).

### 2.1. Production of Antimicrobial Extract

The *Lactiplantibacillus plantarum* LH05 strain was stored at −80 °C and activated in 10 mL of MRS (Man, Rogosa, and Sharpe) medium (Becton Dickinson, Sparks, MD, USA) at 37 °C for 18 h without shaking. Fermentation was conducted in an Erlenmeyer flask containing 100 mL of MRS medium, inoculated with 1% of the activated strain. The flask was incubated anaerobically at 37 °C for 18 h. After fermentation, the culture was centrifuged at 4500 rpm for 15 min at 4 °C to separate the cells. The cell-free crude extract was ultrafiltered with a 10 kDa membrane, followed by a 1 kDa membrane (Millipore, Burlington, MA, USA). The retained fraction from the 1 kDa membrane was lyophilized to concentrate the antimicrobial agent.

### 2.2. Determination of Antimicrobial Activity

The antimicrobial activity of the crude extract was determined using the Kirby–Bauer disk diffusion method. Petri dishes containing Mueller–Hinton agar (Becton Dickinson, Sparks, MD, USA) were inoculated with 1 mL of 1.2 × 10^8^ CFU/mL of each indicator strain (*S. aureus*, *E. coli*, and *S. hominis*). Wells of 0.9 mm were created in the Petri dishes, and 80 µL of the antimicrobial extract was placed in each well. The plates were incubated at 37 °C for 24 to 48 h, and the inhibition zones formed around the wells were measured. Positive inhibition was indicated by clear halos (no growth) around the wells. The antimicrobial activity of the crude extract was also determined using 0.5 cm sensi-discs placed directly on agar containing the indicator strains [15].

### 2.3. Preparation of Biocomposite with Antimicrobial Extract (BAE)

**Figure 1:**
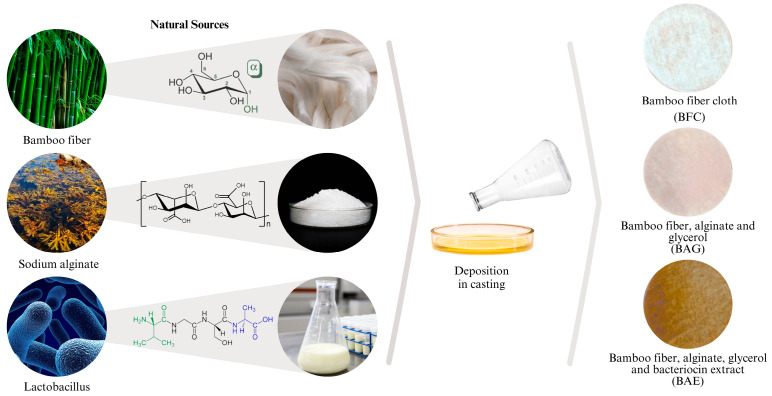
Diagram of the biocomposite production through natural sources and the plate deposition method.

To produce BAE, the solution casting method (as shown in Figure 1) was employed. A solution was prepared in 9 mL of 0.2 M phosphate buffer containing sodium alginate and glycerol at a 1:0.5% ratio. The solution was homogenized by stirring at 600 rpm at 70 °C for 20 min [16]. The lyophilized antimicrobial extract was then dissolved in the final mixture to achieve protein concentrations of 0.54, 1.0, 1.5, 2.2, 3.4, and 4.8 mg/mL, as determined by the Bradford method. The mixture was stirred vigorously for 15 min at room temperature to ensure adequate homogenization. The solutions were validated to determine which one had the minimum inhibitory concentration against the indicator strain *E. coli*. This solution (at 0.54 mg/mL) was used to prepare the biocomposites as follows: the selected mixture was poured onto a bamboo fiber membrane placed in a Petri dish (mold) and left at room temperature until the water evaporated. Once dried, the biocomposites were removed from the mold, and specimens were cut for testing according to standards. Additionally, sensi-discs of 5 mm in diameter were cut for antimicrobial activity assays.

### 2.4. Physico-Mechanical Characterization of the Biocomposite 

Tensile and elasticity tests were performed, and the surface morphology was analyzed using a scanning electron microscope (SEM). Specimens were cut using a CNC laser cutter machine. Tensile tests were performed on an INSTRON 3345 Universal Testing Machine (Instron, Norwood, MA, USA), using a 1 kN load cell with a crosshead speed of 1 mm/min following the ASTM D638-03 standards [17]. Morphological observations were conducted using a HITACHI SEM, model TM-1000 (Hitachi Ltd., Tokyo, Japan), at magnifications ranging from 20 to 10,000×, with a turbo-molecular vacuum pump system, to examine the microstructure and surface morphology of the samples. To assess the contribution of each component to the tensile properties, the tests were conducted on the following materials and biocomposites: (1) bamboo fiber cloth (BFC), (2) bamboo fiber with alginate (BFA), (3) bamboo fiber, alginate, and glycerol (BAG), and (4) biocomposite with antimicrobial extract (BAE, containing bamboo fiber, alginate, glycerol, and antimicrobial extract).

### 2.5. Moisture Absorption

The moisture absorption of the biocomposites was determined using a saturated vapor environment method, as described in ASTM D570 with some modifications [18]. The biocomposites were cut into approximately 1 cm diameter circles, with five samples of each type. The samples were dried in a Luzeren oven at 50 °C for 12 h, cooled in a desiccator, and then weighed until constant weight (*W*_0_) was achieved. The samples were then placed in a desiccator with water, ensuring no direct contact. Weights were recorded as follows: on the first day, every 30 min for 4 h; on the second day, 24 h after the initial recording and 4 h after the second; and from the third day onwards, every 24 h until equilibrium (48 h). Moisture absorption was calculated with Equation (1) using the initial dry weight (*W*_0_) and the sample weight at each measurement time (*W_t_*).

(1) $$Water\;Uptake\;(\%)=\frac{W_{t}-W_{0}}{W_{0}}\times 100,$$

### 2.6. Water Vapor Permeability (WVP)

This test followed ASTM E96 [19], with modifications. Biocomposite disks of approximately 2.5 cm in diameter were dehydrated in a Luzeren oven at 50 °C for 12 h, cooled to room temperature in a desiccator, and weighed to a constant weight. The samples were used as lids for water-filled Teflon cups, ensuring full contact between the sample and the water. The mass loss represented the vapor that permeated through the biocomposite, indicating its permeability. The initial and subsequent weights were recorded at intervals over 48 h, following a method similar to the one reported by Gennadios et al. [20]. The weight was recorded in milligrams. Moisture absorption (Section 2.5) and water vapor permeability (Section 2.6) were tested only in BAG and BAE, as these materials exhibited sufficient mechanical performance and the potential for antimicrobial behavior for wound dressings. Testing could not be performed on the other samples due to their stiffness (BFA) and excessive water loss (BFC).

## 3. Results and Discussion

### 3.1. Evaluation of Antimicrobial Activity of the Extract and BAE

**Figure 2:**
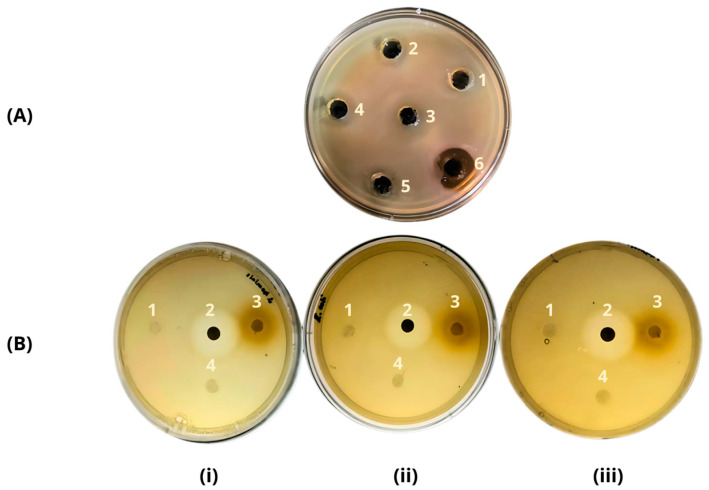
(**A**) In vitro antimicrobial activity (*S. aureus*) of the antimicrobial extract at different protein concentrations: (1) 0.5 mg/mL; (2) 1.0 mg/mL; (3) 1.5 mg/mL; (4) 2.2 mg/mL; (5) 3.4 mg/mL; and (6) 4.8 mg/mL. (**B**) In vitro antimicrobial activity against (**i**) *S. hominis*; (**ii**) *E. coli*; and (**iii**) *S. aureus*. Each plate contains 4 sensi-discs corresponding to (1) BAG; (2) antibiotic control (Novobiocin 5 µg); (3) BAE; and (4) BFC.

*Lactiplantibacillus plantarum* is known to secrete antimicrobial proteins, commonly called bacteriocins, during fermentations [21,22,23,24,25]. This study evaluated the antimicrobial activity of the cell-free extract produced by *Lactiplantibacillus plantarum* HL05 against *Staphylococcus aureus*, *Staphylococcus hominis*, and *Escherichia coli*. Figure 2A shows results for *S. aureus*. The antimicrobial effect of the crude extract resulted in increasing inhibition zones as the concentration increased. The largest inhibition halo (approximately 20 mm) was observed at the maximum protein concentration (4.8 mg/mL) across all strains. The minimum inhibitory concentration was at the lowest protein concentration (0.54 mg/mL), and this concentration was used to produce the BAE samples.

The antimicrobial activity of BAE is shown in Figure 2B for all strains. Inhibition halos were observed for all three strains, suggesting the presence of bacteriocins (plantaricins) in the extract. Previous studies report the ability of lactic acid bacteria like *L. plantarum* to produce antimicrobial compounds that control and inhibit the growth of competing bacteria. *L. plantarum* produces several bacteriocins, such as plantaricin 423, with enhanced activity due to the synergistic peptide interactions (plantaricin E with plantaricin F and plantaricin J with plantaricin K) leading to an increased antimicrobial activity against pathogens. Two accepted mechanisms explaining the antimicrobial activity of bacteriocins are their ability to disrupt lipids in the cell membranes of pathogens and their effect on bacterial metabolism by affecting nucleic acids and proteins [21,22,23,24,25,26,27].

Clinically, *S. aureus*, *S. hominis*, and *E. coli* are common wound pathogens, complicating and delaying patient healing [28]. The biocomposite incorporating the antimicrobial extract from *L. plantarum* LH05 shows promise as a therapeutic agent in wound treatment, with direct antimicrobial activity and its potential to modulate the immune response and promote an effective healing environment. However, further studies are needed to understand its action mechanism and efficacy in specific clinical applications, such as skin wounds [29].

### 3.2. Physico-Mechanical Properties: Elastic Modulus

The mechanical (tensile) tests demonstrate the contribution of all materials to the biocomposite: (1) bamboo fiber cloth (BFC), (2) bamboo fiber with alginate (BFA) as matrix, (3) bamboo fiber, alginate, and glycerol (BAG) as plasticizer, and (4) biocomposite with antimicrobial extract (BAE), also containing bamboo fiber, alginate, and glycerol.

**Figure 3:**
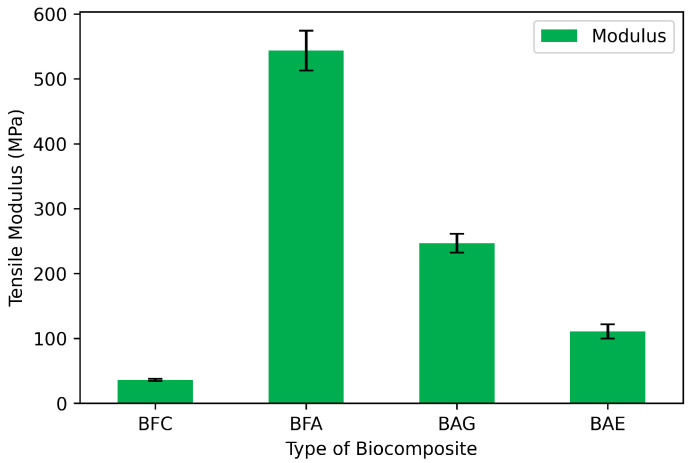
Elastic modulus of the biocomposites: bamboo fiber cloth (BFC); bamboo fiber with alginate (BFA); bamboo fiber, alginate, and glycerol (BAG); and biocomposite with antimicrobial extract (BAE).

Figure 3 shows that BFC exhibited a low elastic modulus, indicating low rigidity, which is consistent with the fibrous nature of the material (cloth), offering physical flexibility due to its chemical composition and the morphology of lignocellulosic fibers [30,31]. Natural fiber-based biocomposites, such as bamboo fiber, are flexible and biodegradable structural materials. They are also natural, abundant, and have a lower carbon footprint compared to petroleum-derived composites [32]. However, they lack the liquid retention or functionalization required for wound treatment applications.

Adding alginate to bamboo fiber cloth (BFA) increased the elastic modulus significantly (from 36 to 544 MPa), indicating that alginate acts as a polymer matrix, providing the mechanical stability required for medical use [33]. However, excessive rigidity can limit deformability and, consequently, its ability to adapt to the patient’s skin and anatomy. The addition of glycerol to the bamboo fiber–alginate composite (BAG) as a plasticizing agent aimed to control rigidity and reduce the elastic modulus, resulting in a decrease from 544 to 247 MPa. Incorporating the antimicrobial extract (BAE) increased the plasticizing effect, thus reducing the elastic modulus to 111 MPa. This is because the bioactive agent also interposes between the alginate matrix chains, preventing secondary interactions among them [34]. The BAE formulation provides mechanical advantages to the material, achieving controlled rigidity akin to bamboo cellulose, while maintaining a defined structure (continuous phase and dispersed phase). This can enhance other mechanical properties without compromising the biodegradable nature of the materials (Figure 3).

Previous studies have shown that the elastic modulus of dressings largely depends on factors such as fiber content and the adhesion between the fiber and the polymer matrix [34]. Bamboo fibers, composed of cellulose, hemicellulose, and lignin, provide flexibility and strength, making them suitable for structural applications [35]. It is important to highlight that each component of BAE contributes to its mechanical stability, crucial for potential biomedical applications.

Sodium alginate was shown to be effective in wound dressing fabrication, not only enhancing mechanical properties, but also serving as a drug delivery system that facilitates wound healing due to its hydrophilic nature and high water absorption capacity. These systems can retain water and bioactive substances with antimicrobial activity, releasing them gradually in a controlled manner [36].

### 3.3. Tensile Strength

**Figure 4:**
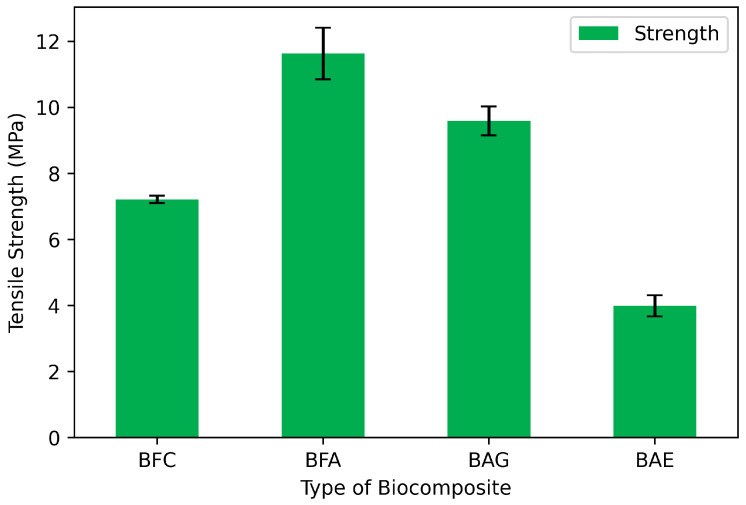
Tensile strength of the biocomposites: bamboo fiber cloth (BFC); bamboo fiber with alginate (BFA); bamboo fiber, alginate, and glycerol (BAG); and biocomposite with antimicrobial extract (BAE).

The tensile test results, shown in Figure 4, reveal the maximum mechanical strength of the materials. As for the elastic modulus, alginate provided greater strength and stability to the biocomposite compared to the material made solely with bamboo fiber cloth (BFC), which exhibited a strength of 7.6 MPa. Adding alginate to BFC increased the strength to 12 MPa. Although this value is the highest obtained, it is not optimal due to its high rigidity (Figure 3). Therefore, adding a plasticizer (to control rigidity) and the antimicrobial extract (for functionalization) was necessary. Consequently, the tensile strength decreased from 12 MPa to 10 MPa and 4 MPa for BAG and BAE, respectively. Despite this reduction, the final value is similar to BFC. The literature reports that components such as glycerol, acting as plasticizers in biopolymer films, can affect strength and other properties like toughness and deformability.

This effect is attributed to the lubricating effect of plasticizers, which, by preventing intermolecular interactions, facilitate the reciprocal movement of the macromolecular chains that make up the biopolymer [37]. These properties have been widely reported in other biopolymers such as poly(lactic acid), which, due to its rigid nature, has been blended with triethyl citrate [38], polyethylene oxide, various vegetable oils, and lipid derivatives, as well as glycerol, to name but a few [39].

### 3.4. Elongation at Break

**Figure 5:**
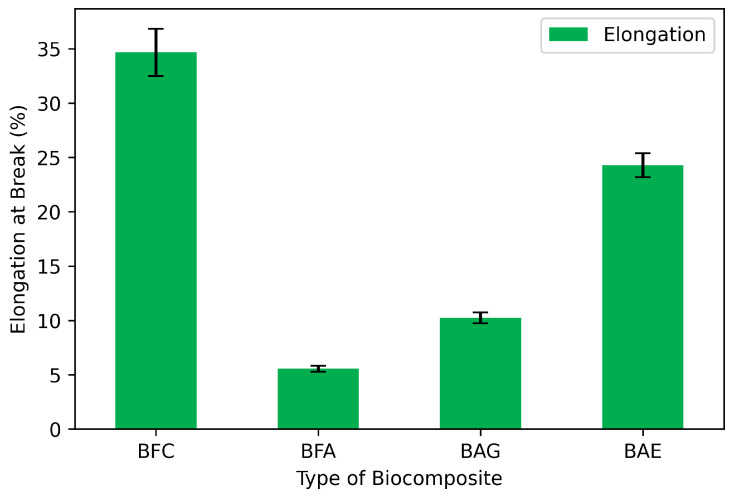
Elongation at break of the biocomposites: bamboo fiber cloth (BFC); bamboo fiber with alginate (BFA); bamboo fiber, alginate, and glycerol (BAG); and biocomposite with antimicrobial extract (BAE).

Deformation at break measures how much the composites deform just before fracturing. As shown in Figure 5, BFC exhibits a greater capacity for deformation due to its woven fibrous structure. This deformation behavior is also evidenced by [40], who developed a biocomposite with different types of woven cellulose. However, this fibrous structure lacks a defined matrix or continuous phase, needed to retain active compounds for stable functionalization. The BFA biocomposite exhibited a decrease in deformation at break from 37% (BFC) to 6% (BFA), due to the increase in rigidity (modulus) previously discussed. Adding glycerol (BAG) and the antimicrobial extract (BAE), significantly increased deformation capacity to 24%, close to the BFC values but with a firmer and more functional structure that allows for material functionalization.

Studies with biopolymers have shown that adding plasticizers such as oils or nanoparticles (e.g., silicon-based) [41], improves mechanical properties by reducing cohesion within the film structure and weakening intermolecular forces. This results in improved properties such as flexibility and polymer deformation, while providing a hydrophilic polymer matrix with better barrier properties. Moreover, sodium alginate’s hygroscopic characteristics contribute to this film’s hydrophilic property [42]. Flexibility is desirable for films used therapeutically in wound or burn dressings, as sufficient mechanical strength and deformation capacity will maintain their integrity against external stresses during handling, cleaning, removal from the skin, and adapting to the patient’s natural movements [43].

**Figure 6:**
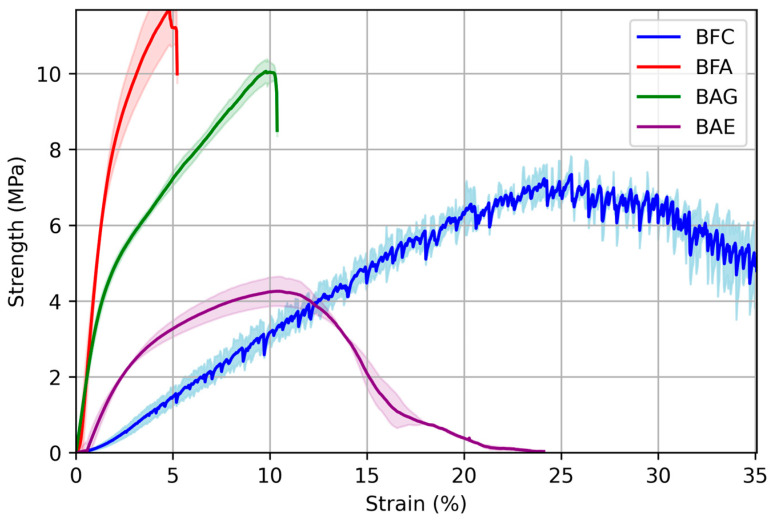
Stress–strain curves of the biocomposites: bamboo fiber cloth (BFC); bamboo fiber with alginate (BFA); bamboo fiber, alginate, and glycerol (BAG); and biocomposite with antimicrobial extract (BAE). The shaded area represents the standard deviation.

The mechanical performance of the biocomposites is jointly appreciated in the stress–strain curves in Figure 6. The BFC stress–strain curve demonstrates high elasticity and deformation capacity but low mechanical stability, evidenced by erratic behavior during stress transmission due to fiber separation, which complicates its potential use as a dressing.

Adding alginate and subsequently the plasticizer improved the material’s toughness, allowing greater composite deformation. Although the maximum strength decreased, more energy was required to break the composite. This combination of materials provides the necessary properties for functionalized medical devices [44]. Similar effects have been observed in hyperbranched and dendritic polymers or block copolymers using glycerol as a plasticizer for biomedical applications, with the advantage of water solubility and biocompatibility [45].

Incorporating alginate as a polymer matrix, glycerol as a plasticizer, and the antimicrobial extract for biocomposite functionalization (BAE) results in a material with a plastic behavior similar to bamboo fiber, maintaining comparable strength and toughness but with greater mechanical stability. This is reflected in higher deformation capacity and especially in the ability to be functionalized while having a more uniform structure.

### 3.5. Morphology (SEM)

**Figure 7:**
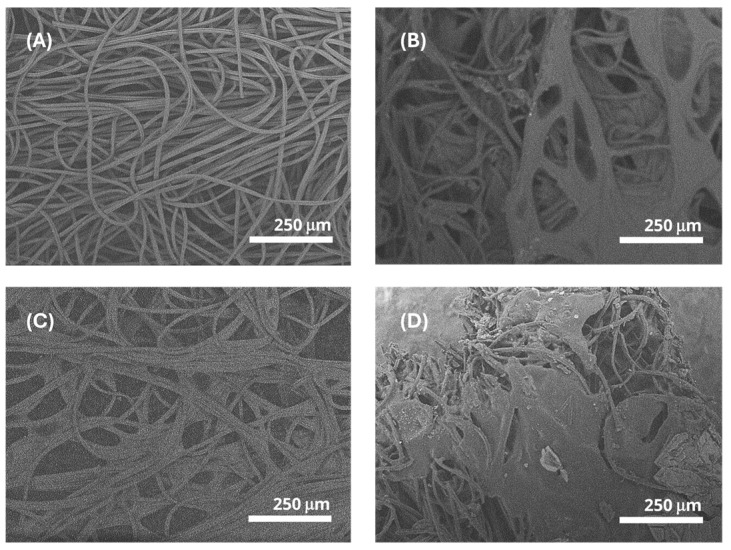
SEM micrographs at 200× of the biocomposites: (**A**) BFC; (**B**) BFA; (**C**) BAG; and (**D**) BAE.

SEM observations were performed to analyze the interface behavior between bamboo fiber and the entire polymer matrix. Figure 7 presents micrographs of BFC, showing its woven cellulose fiber structure without a continuous medium or matrix (Figure 7A). The micrographs of the BFA and BAG biocomposites in Figure 7B,C show bamboo fibers bound in a continuous medium (alginate), providing stability that is consistent with the tensile test results. Alginate, functioning as a fiber-binding agent, likely gives the biocomposites the ability to endure greater stresses with less risk of breakage.

In the micrograph of the functionalized biocomposite (BAE, Figure 7D), a greater fiber coating is observed, aligning with the previously discussed mechanical properties. Therefore, it is confirmed that bamboo fibers create discontinuities in the material, corrected by adding other biocomposite components, including the antimicrobial extract. Similar results were described for the mechanical properties and SEM morphology of bamboo fiber in a polyester resin composite [46]. Notably, all biocomposites exhibited porosity, important for water interaction, a key requirement for an antimicrobial dressing that promotes moist wound healing [47]. BAE shows a higher degree of fiber coating (lower porosity), which may influence water or exudate interaction.

### 3.6. Moisture Absorption and Water Vapor Permeability (WVP)

**Figure 8:**
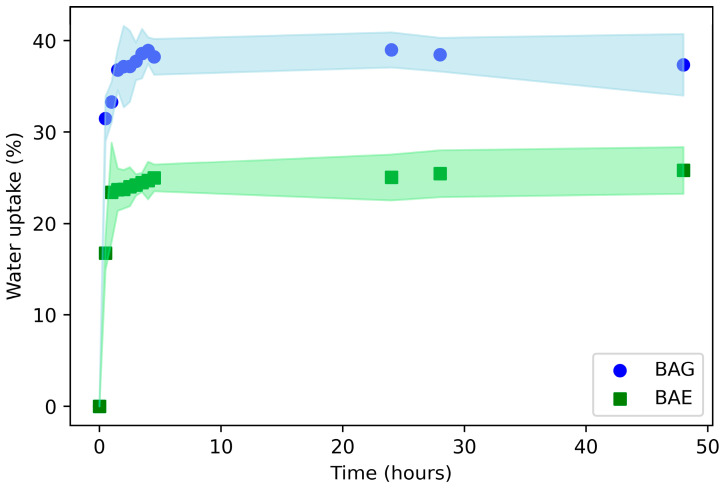
Moisture absorption of the biocomposites BAG and BAE. The shaded area represents the standard deviation.

Figure 8 shows that BAG samples had a moisture absorption of approximately 38%, higher than the BAE biocomposites, which had an absorption of around 25%. This is likely because the film without the antimicrobial extract consists of highly hydrophilic materials such as bamboo lignocellulosic fibers and sodium alginate. Similar results were reported by [48], who found that adding sodium alginate to a bacterial cellulose membrane produced a material with greater moisture retention capacity.

The reduced water retention capacity in BAE films is due to the addition of a less hydrophilic material (antimicrobial extract), which can form hydrogen bonds with the hydroxyl groups of cellulose and alginate, preventing them from subsequently interacting with water [49]. Additionally, SEM micrographs revealed a higher degree of hydrophilic material coverage by the antimicrobial extract, which reduces the area available for water interaction (lower porosity). Similar trends have been observed in alginate-based films and other polysaccharides reinforced with various compounds. These interact with -OH groups (via hydrogen bonds or covalent bonds due to crosslinking), reducing moisture retention, for example, cellulose nanocrystals [49], polyethylene glycol [50], or lignin [51]. The result is a more stable biocomposite that can retain a significant amount of water but is less sensitive to humidity changes (dimensional stability), favoring healing in a moist environment and proper wound exudate retention [47].

**Figure 9:**
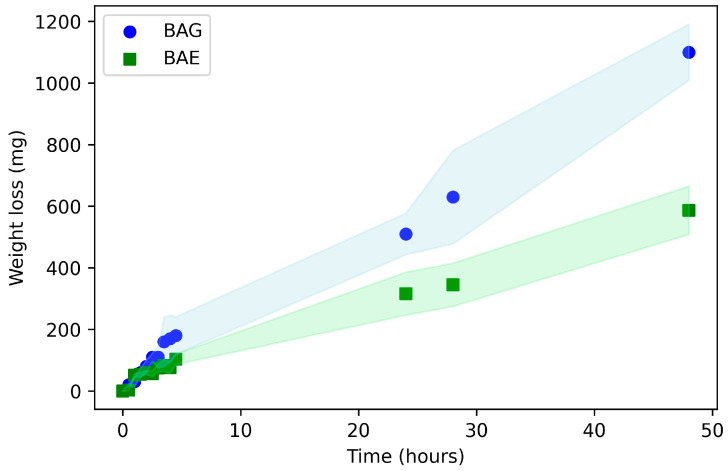
Water vapor permeability (water weight loss through the film) of the biocomposites BAG and BAE. The shaded area represents the standard deviation.

On the other hand, Figure 9 shows the weight loss over time during the water vapor permeability test using the wet cup method. For both biocomposites, the water loss rate was linear for the first hour, attributed to the time it takes for the biocomposite to reach equilibrium. From that point until the end of the 48 h test duration, the linearity of water vapor weight loss over time was maintained, indicating that the biocomposites exhibited a steady-state permeability rate [20]. As expected, the biocomposite containing the antimicrobial extract (BAE) showed lower permeability (587 mg) compared to the bamboo fiber and glycerol biocomposite (BAG), which exhibited a water mass loss of 1100 mg. This difference is linked with the physico-mechanical properties regarding water absorption and SEM-observed morphology, indicating that the BAE biocomposite’s functionality is more stable while maintaining its desired hydrophilic nature for medical applications. Based on the results obtained (Figure 8 and Figure 9), BAE showed suitable moisture retention and vapor transmission rates, helping maintain optimal moisture at the wound–dressing interface while preventing the accumulation of secretions and excess fluids. This not only supports antimicrobial control but also promotes wound healing [47].

## 4. Conclusions

Bamboo fiber, alginate, and antimicrobial extract biocomposites offer a promising alternative for treating skin wounds due to their physico-mechanical properties, chemical composition, biodegradability, and environmental compatibility. The combination of these components produces a biocomposite material with synergistic and specific properties: the flexibility of bamboo fiber, the ability to retain an antimicrobial active compound, the stability and strength of alginate, and the functionality and flexibility of a plasticizer, while maintaining appropriate water retention and permeability. The result is a material potentially suitable for wound dressing applications. However, in vivo studies are essential to validate the application and functionality of the designed biocomposite. In vivo studies are currently underway.

## Data Availability

The raw data supporting the conclusions of this article will be made available by the authors on request.
